# Osteopetrosis in two siblings: two case reports

**DOI:** 10.1186/s13104-016-1869-x

**Published:** 2016-01-29

**Authors:** Satish Yadav, Shiv Chalise, Shipra Chaudhary, Gauri Shankar Shah, Mukesh Kumar Gupta, Om Prakash Mishra

**Affiliations:** Department of Pediatrics and Adolescent Medicine, B. P. Koirala Institute of Health Sciences, Dharan, Nepal; Department of Radiology, B. P. Koirala Institute of Health Sciences, Dharan, Nepal; Department of Pediatrics, Institute of Medical Sciences, Banaras Hindu University, Varanasi, India

**Keywords:** Osteopetrosis, Deafness, Radiological appearance

## Abstract

**Background:**

Osteopetrosis is a rare inherited metabolic bone disorder characterized by extensive sclerosis of skeletons, visual and hearing impairment, hepatosplenomegaly and anemia. It has two major clinical forms: the autosomal dominant adult (benign) form is associated with milder symptoms often appearing in later childhood and adulthood whereas the autosomal recessive infantile (malignant) form has severe presentations appearing in very early childhood, if untreated, is typically fatal during infancy or early childhood. A rare autosomal recessive (intermediate) form is present during childhood with some signs and symptoms of malignant osteopetrosis. Diagnosis is mainly based on clinical and typical generalized increase in bone density.

**Case presentation:**

The two siblings of Indo-Aryan ethnicity, aged five and 8 years, were admitted with irregular low grade fever and gradually increasing abdominal mass for last 3 years. They also had history of hearing loss. On examination, the patients were found pale with poor nutritional status, short stature, frontal bossing and splenomegaly. We made a clinical diagnosis of hemolytic anemia and investigated accordingly. Peripheral Blood Smear was suggestive of leucoerythroblastic picture in both the siblings. We extended our investigations and radiological survey revealed generalized increase in bone density which was consistent with osteopetrosis.

**Conclusion:**

Osteopetrosis is a rare disease transmitted by autosomal dominant or recessive inheritance having variable penetrance. We report here milder form of disease in the two siblings having typical clinical features in the form of anemia, hepatosplenomegaly and hearing loss. Diagnosis was confirmed by typical generalized increase in bone density in both the patients.

## Background

The term osteopetrosis is derived from the Greek ‘osteo’ meaning bone and ‘petros’, for stone. It is also known as ‘marble bone disease’ and ‘Albers-Schönberg disease’, after the German radiologist reported first description of the condition in 1904 [1]. This disorder includes impaired osteoclast function and marked increase in generalized bone density.

The estimated prevalence of osteopetrosis is 1 in 100,000–500,000 births [2]. It is present as two major clinical forms-the autosomal dominant adult (benign) form is associated with a very few or no symptoms and the autosomal recessive infantile (malignant) form is typically fatal during infancy or early childhood, if left untreated. Another milder recessive (intermediate) disease has less severe presentation [3]. This is also presents in milder form due to deficiency of carbonic anhydrase II enzyme in some patients.

## Case presentation

The two siblings (both males) of Indo-Aryan ethnicity, aged 5 and 8 years, were admitted to the Department of Pediatrics and adolescent medicine with history of irregular low grade fever and gradually increasing mass in the abdomen for the last 3 years. They had history of hearing loss. There was no history of previous blood transfusion, family history of similar disease, consanguinity or any hematological disease and vision loss. Antenatal and natal periods were uneventful. There was another sibling who was 12 years old and healthy.

On examination, the patients were found to be moderately pale with poor nutritional status, short stature, frontal bossing and malformed craniofacial appearance. The younger sibling had splenomegaly of 10 cm below left sub-costal margin in its axis but no hepatomegaly. The older sibling had both hepatomegaly of 3 cm and splenomegaly of 8 cm. Brain stem evoked response audiometry revealed hearing loss in both the patients. Ophthalmological examination was normal except nystagmus in the younger sibling.

Hemogram revealed anemia in both the siblings. Hemoglobin levels were 7.5 gm/dl in younger and 5.6 gm/dl in older sibling (ref range: 11.5–13.5 gm/dl). Peripheral blood smear was suggestive of leucoerythroblastic picture. Other laboratory investigations yielded the following: serum total calcium 8.8 and 9.1 mg/dl (ref range: 8.7–10.3 mg/dl), phosphorous 4.3 and 4.5 mg/dl (ref range: 3.2–6.3 mg/dl) and alkaline phosphatase were 235 and 345 U/L (ref range 86–315 U/L) respectively in younger and older siblings. Malaria and Kala azar were ruled out by serological test as the patients were from endemic zone. The exact cause of irregular low grade fever could not be elucidated except urine routine microscopy showed 5–7 white blood cell which was treated with oral antibiotic. On further visit, both the siblings were afebrile.

**Fig. 1:**
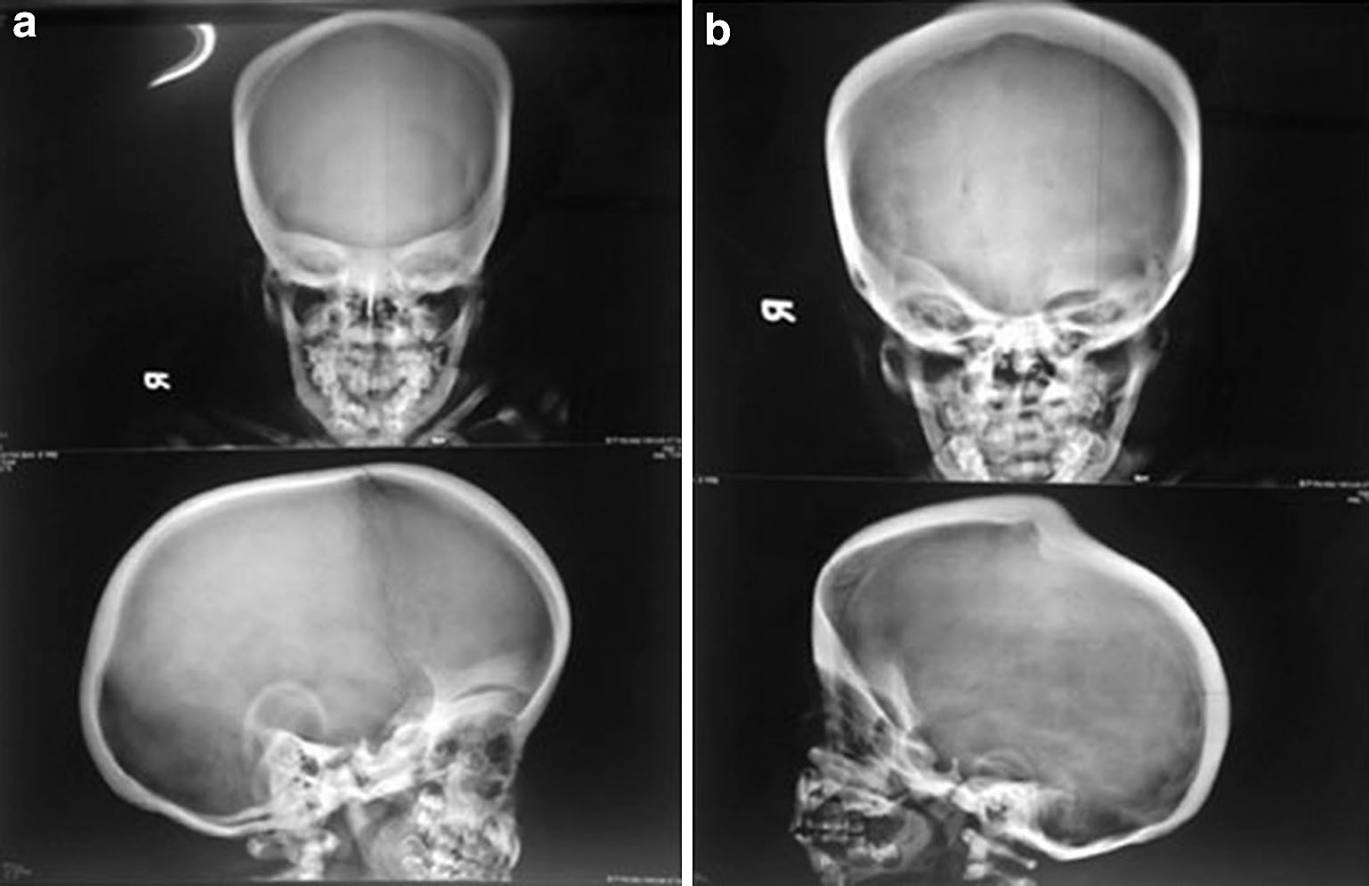
Calvarial thickening with diffused increase in density of the skull bones with poor development of sinuses (**a** 5 years, **b** 8 years)

**Fig. 2:**
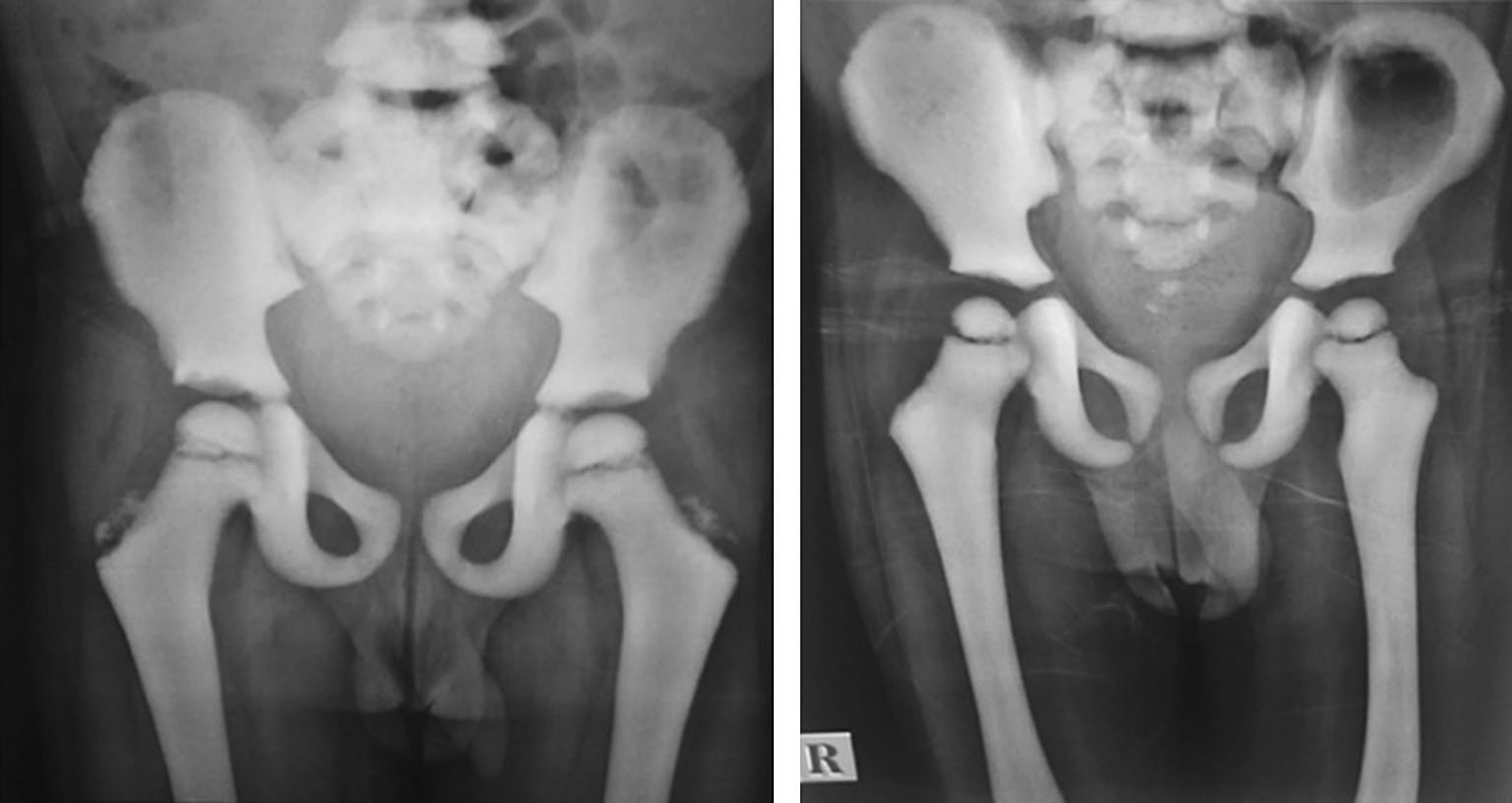
Homogeneous increased density of bone with little differentiation between cortex and medulla in long bones and pelvis (**a** 5 years, **b** 8 years)

**Fig. 3:**
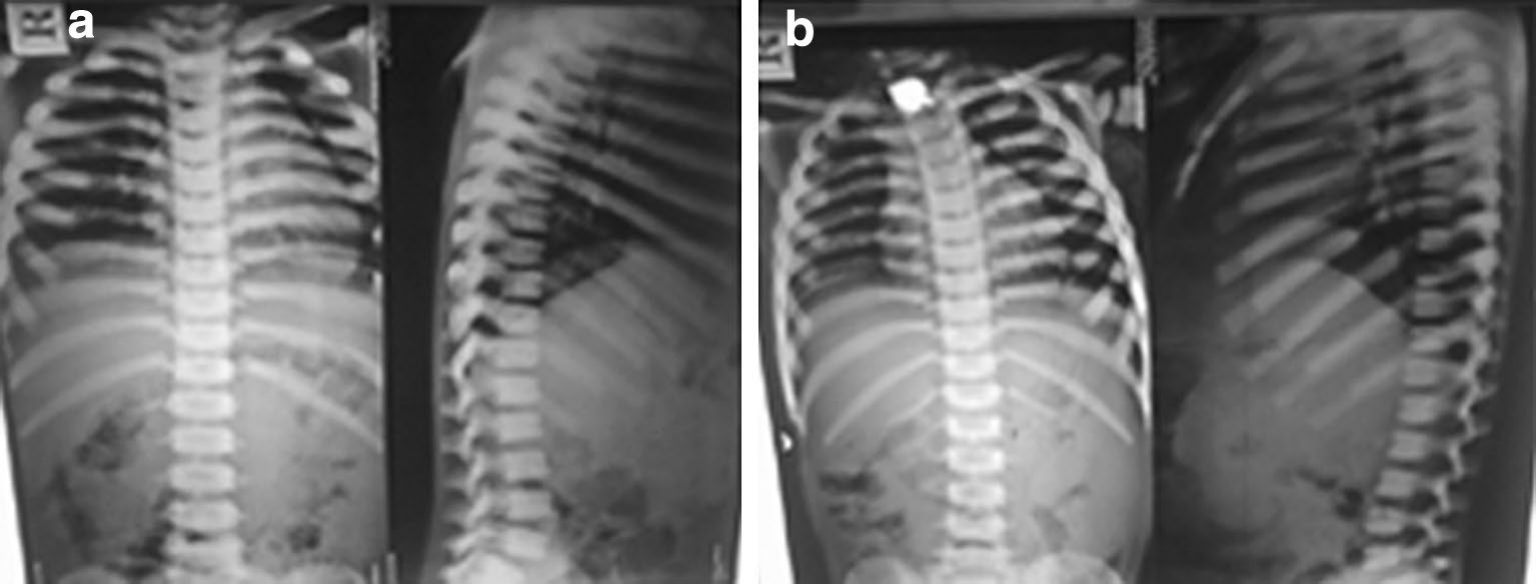
Sclerosis of vertebral endplates resulting in “sandwich vertebrae” appearance and increased density of ribs (**a** 5 years, **b** 8 years)

Radiological features of both the siblings revealed calvarial thickening with diffused increase in density of the bones with poor development of sinuses (Fig. 1a, b). There was homogeneous increased density of bones with little differentiation between cortex and medulla in long bones and pelvis (Fig. 2a, b). X-ray of vertebrae showed sclerosis of vertebral endplates resulting in “sandwich vertebrae” appearance and increased density of ribs (Fig. 3a, b).

Treatment which was given to both the siblings was conservative and supportive in the form of blood transfusion and regular visit for ophthalmic and dental surveillance as the disease pattern was less severe type.

## Discussion

Clinical manifestations of osteopetrosis are highly variable, ranging in severity from asymptomatic to fatal course. Our two sibs had clinical features consistent with mild recessive (intermediate) form of disease supported by classical radiological findings. This form is more severe than the adult form but less severe than the malignant infantile form. The main features are short stature, frontal bossing with dysmorphic craniofacial appearance, pallor, splenomegaly and hearing loss. Short stature is due to impaired longitudinal growth and dysmorphic craniofacial appearance is caused by macrocephalus and bossing of forehead [4]. Anemia in osteopetrosis is leukoerythroblastic in type which was present in both the siblings [5].

The infantile malignant form is characterized by severe clinical features manifesting in the first few months of life. The presenting features are fractures and osteomyelitis, short stature, typical facial appearance due to macrocephaly and frontal bossing [6]. The expanding bone can narrow nerve foramina involving cranial nerves resulting in blindness, deafness, and facial palsy [7]. The abnormal expansion of bone interferes with medullary haematopoiesis resulted in life-threatening pancytopenia and extramedullary haematopoiesis which may lead to enlargement of liver and spleen. This form is fatal in majority of cases within the first 5 years of life.

The mainstay of diagnosis largely depends on the radiographic appearance of the skeleton. There are diffused osteosclerosis affecting the skull, spine, pelvis and appendicular bones, cortical thickening with medullary encroachment, poorly pneumatized paranasal sinuses and “Sandwich” appearance of vertebrae due to alternating sclerotic and radiolucent transverse metaphyseal lines [4]. There is no demarcation of cortex and medulla in the long bones.

Hematopoietic stem cell transplantation (HSCT) is the only treatment that can offer cure to such patients. HSCT using HLA identical donors results in 73 % 5 years disease free survival [8]. Interferon gamma 1b (IFNγ1b) treatment has been tried in patients with osteopetrosis variants unresponsive to HSCT or as a bridging therapy to transplantation. It has been reported to result in improvement in immune function, increased bone resorption and increase in bone marrow space [9–11].

## Conclusion

Though osteopetrosis is a rare disease, one can consider in children presenting with unexplained anemia and hepatosplenomegaly and radiological survey is required to confirm the diagnosis.

## Consent

Written informed consent was obtained from the patients’ parents for publication of this case report and any accompanying images.

## Abbreviations

HSCT: hematopoietic stem cell transplantation
IFNγ1b: interferon gamma 1b
